# Effects of Unusual Gate Current on the Electrical Properties of Oxide Thin-Film Transistors

**DOI:** 10.1038/s41598-018-32233-4

**Published:** 2018-09-17

**Authors:** Jinwon Lee, Keon-Hee Lim, Youn Sang Kim

**Affiliations:** 1 Program in Nano Science and Technology, Graduate School of Convergence Science and Technology, Seoul National University, 1 Gwanak-ro, Gwanak-gu, Seoul 08826 Republic of Korea; 2 Advanced Institute of Convergence Technology, 145 Gwanggyo-ro, Yeongtong-gu, Suwon 16229 Republic of Korea

## Abstract

The wide research and development on oxide thin-film transistors (TFTs) have led to considerable changes in mainstream technology in various electronic applications. Up to now, much research has been focusing on enhancing the performance of oxide TFTs and simplifying fabricating process. At the stage of research and development in the oxide TFT, unexpectedly high gate current phenomena have been continuously reported by several groups, but the origins have not been yet studied in detail. The unusual gate current interferes with the conductance of the oxide TFT, which makes it difficult to interpret the performance of the TFT. Here we present the origin and control factors of the unconventional gate currents flow in the oxide TFT. The gate current is due to the conduction of electrons through trap sites in insulators, and the current is sophisticatedly controlled by the structural factors of TFT. Furthermore, the gate current flows only in one direction due to the charge state of the oxide semiconductor at the interface with the insulator. We also demonstrate that the vertical current path functions as a diode unit can protect the TFT from unintended gate electrostatic shock.

## Introduction

A thin-film transistor (TFT), which is one type of the metal-oxide-semiconductor field-effect transistor (MOSFET), is a key component for thin-film technology and its most important application is a switching element on display products. Recently released display products have used oxide semiconductor TFTs with high electron conductance in order to realize high resolution and transparency^1–4^. Most conventional oxide TFTs have a bottom gate structure consisting of a gate electrode, dielectric insulator, oxide semiconductor, and source and drain electrodes^5–7^. The oxide semiconductor active layer of the oxide TFTs designed for commercialization should be patterned to have a size similar to that of the source and drain electrodes in order to avoid fringe electric fields or parasitic capacitances^8–10^. These days many research and development on the oxide TFTs have focused on improving manufacturing processes including solution coating and printing technologies^11–18^. The oxide TFTs in such a research stage are often fabricated with large pattern sizes or without a patterning process of the oxide semiconductor layer for their ease of research and manufacture^19,20^. Theoretically, gate leakage currents are completely blocked in conventional bottom gate structure TFTs regardless of the area size of active junction because a thick gate dielectric layer electrically insulates a gate electrode from a source electrode and a drain electrode. However, unconventional results of high gate leakage currents flowing in only one direction in the oxide TFTs with a large area of oxide semiconductor layer have been steadily reported by many groups^21,22^. The unconventional gate leakage currents are normally reduced after patterning process in their reports^23^; however, the exact origins and the details of the uni-directional gate leakage current are still unknown.

Recently we have studied that the unusual high vertical current flows through a relatively-thick insulator film in metal – insulator – oxide semiconductor structures. We have also verified that the magnitude and direction of the unconventional vertical current is also controlled by adjusting material properties of the top semiconductor electrode^24^. Herein, we have studied the origins of the unconventional vertical current through insulator films in more depth and the effect of the vertical current on the electrical performance of conventional oxide semiconductor TFT devices. First, we have approached probabilistically that the junction area between the top electrode and insulator layer affects the leakage current flow in normal metal/thick insulator/metal (MIM) structures. Based on the effect of junction area, the effects of gate currents on conductance characteristics of bottom gate oxide TFT devices having large active layer area are investigated. We have, then, demonstrated that the gate current flowing in only one direction can be utilized as an electrostatic discharge diode (ESD) path to protect the oxide TFT from unexpected gate electrostatic shocks.

## Results

### **I**nterference to oxide TFT performance of abnormal gate current

**Figure 1:**
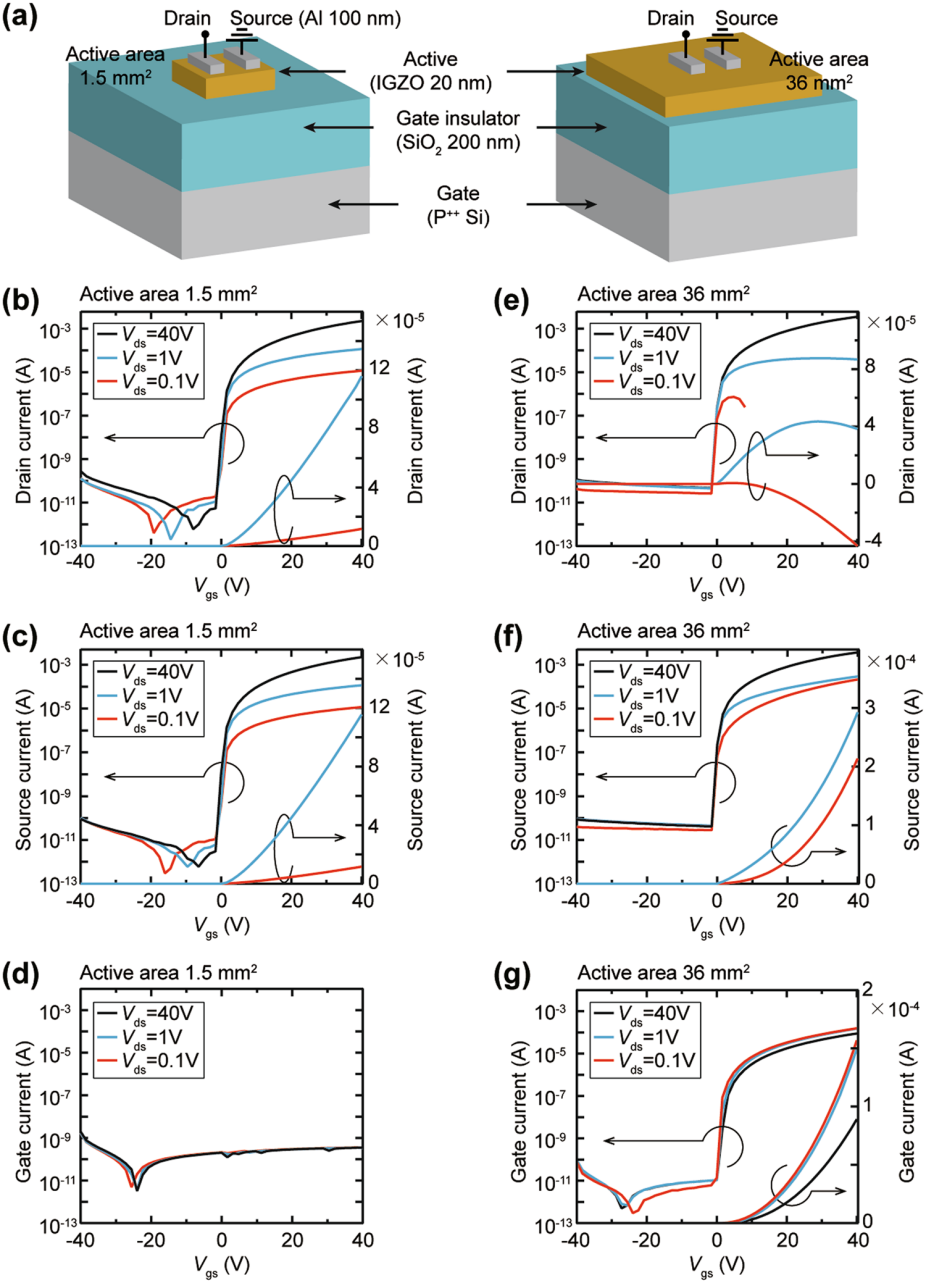
The electrical characteristics of the two oxide TFTs with different active layer area. (**a**) Schematic structures of the TFTs with different active layer area of 1.5 mm^2^ and 36 mm^2^. (**b**–**d**) Electrical currents measured at (**b**) drain, (**c**) source and (**d**) gate electrodes in the TFT with 1.5 mm^2^ IGZO active layer. (**e**–**g**) Electrical currents measured at (**e**) drain, (**f**) source and (**g**) gate electrodes in the TFT with 36 mm^2^ IGZO active layer.

To confirm an effect of the junction area size on the gate current phenomenon in bottom gate structured oxide TFTs, we compared oxide TFTs consisting of oxide semiconductor active layers with different areas (Fig. 1a). Highly doped p-type silicon (P^++^ Si) was used as a substrate and a gate electrode, and thermally oxidized SiO_2_ with a thickness of 200 nm was used as a gate insulator. The IGZO (In:Ga:Zn:O = 1:1:1:4 at%) oxide semiconductor with a thickness of 20 nm was deposited on the SiO_2_ using an RF magnetron sputtering system, and the IGZO layer was patterned on its sides to prevent inadvertent lateral contact with the gate electrode. Source and drain electrodes of Al metal with a thickness of 100 nm were formed on the oxide semiconductor film using a thermal evaporator, and a channel width and length of the electrodes are 1000 μm and 50 μm, respectively. In the oxide TFT with an active area of 1.5 mm^2^, the oxide semiconductor layer has a similar area to the source and drain electrode area, whereas the compared oxide TFT has an IGZO area as wide as 36 mm^2^. The electrical characteristics for the both TFTs were measured using a probe station at a source-drain voltage (*V*_ds_) of 0.1 V to 40 V with the source-gate voltage (*V*_gs_) sweeping from −40 V to 40 V. The transfer curves for the TFT with 1.5 mm^2^ IGZO exhibit typical n-type oxide semiconductor TFT characteristics^25,26^. A drain current (*I*_d_) at a positive *V*_gs_ region, which is measured at the drain electrode, increases proportionally as the *V*_ds_ increases from 0.1 V to 40 V, and the off-state currents at a negative *V*_gs_ range remain very low levels of 10 pA (Fig. 1b). Source current value (*I*_s_), which is measured at the source electrode, equals to amounts of the drain currents (*I*_d_) escaped from the drain electrode (Fig. 1c). The gate currents (*I*_g_), commonly referred to as a leakage current, are negligible under ~pA level at the entire *V*_gs_ range (Fig. 1d). Therefore, it is verified that the SiO_2_ gate insulator layer blocks well the transportation of any electrical charge carriers including electrons, holes, and ionized carriers between the gate and the source or drain electrodes. The results demonstrate that the operation of the oxide TFT with 1.5mm^2^ IGZO active layer agrees well with the basic TFT operating principle that the channel conductivity is controlled in proportion to the *V*_gs_.

The drain currents of the TFT with 36 mm^2^ IGZO area show completely different patterns with the TFT with 1.5 mm^2^ IGZO film. In the off-state under a negative *V*_gs_ range, the *I*_d_ are almost the same as the *I*_d_ for the TFT with 1.5 mm^2^ IGZO; however, the on-state *I*_d_ at a positive *V*_gs_ range appears to be distorted in a log scale, and the degree of distortion becomes serious as the *V*_ds_ decreases toward 0.1 V (Fig. 1e). The drain current curves in a linear axis show that the drain current begins to decrease from a specific *V*_gs_. Furthermore, the *I*_s_ values are much higher than the *I*_d_ values flowing out from the drain electrode (Fig. 1f), which implies that another current flows in or out at both the drain and source electrodes. The significant difference of the *I*–*V* characteristics with the oxide TFT having 1.5 mm^2^ IGZO layer is clearly shown in the gate current curves. The gate current of the oxide TFT having 36 mm^2^ IGZO active layer hardly flows in the negative *V*_gs_ region but begins to increase sharply at *V*_gs_ = 0 V, and a very high current of 1.5 × 10^−4^ A flows at *V*_gs_ = 40 V (Fig. 1g). The unusual gate current characteristic indicates that electrical charge carriers are actively transported through the 200-nm SiO_2_ layer between the gate electrode and the drain or source electrodes at a positive *V*_gs_. This result is in contradiction with the result of the TFT with a 1.5 mm^2^ IGZO in which 200-nm SiO_2_ maintains good insulating property and completely blocks the leakage current; thus, it can be deduced that the contact size at the active/gate insulator junction directly affects the gate leakage current flow.

### Origins of the uni-directional gate current in the oxide TFT

In order to clarify the influence of the electrode contact area on the current flowing through SiO_2_ of 200 nm thickness, the vertical currents were compared in P^++^ Si/200-nm SiO_2_/Al (MIM) structure and P^++^ Si/200-nm SiO_2_/IGZO (MIS) structure having various area of the Al and IGZO top electrodes. The area of the Al and IGZO electrodes were varied from 0.2 mm^2^ to 28.26 mm^2^ (circle shapes with diameters from 0.5 mm to 6 mm), and we measured 40 devices for each electrode area. There are no vertical current flows in the MIM with a 0.2 mm^2^ Al electrode. However, the vertical current starts to flow as the Al area increases, and most of the devices having the Al electrode of 28.26 mm^2^ area allows a high vertical current over 10^−6^ A (Fig. S1). The increase in the vertical current through the 200-nm SiO_2_ due to the increase of the contact area is also confirmed in the MIS devices using IGZO top electrode. In the MIS structures, as in the result of a gate current flowing in the TFT with large IGZO active layer, vertical currents flow very little in a negative voltage and high vertical currents flow in only one direction in a positive voltage region (Fig. S2). Cumulative distribution graphs of the vertical current values measured at 1 V in the MIM structures and measured at 10 V in the MIS structures clearly show that the vertical current through the SiO_2_ layer increases sharply as the contact area of the electrode increases (Fig. S3). Because the thickness of SiO_2_ is 200 nm, it is very difficult for the conduction to be caused by quantum tunneling of electrons, and no electric breakdown occurs, as confirmed by the *I*–*V* characteristics of the MIS device. Thus, it can be reasonably deduced that the larger electrode area increases the probability of overlap with the trap sites inherently present inside the SiO_2_ layer, and electrons are injected and transmitted through these overlapped trap sites^27–30^. In addition, this statistical dependence on the junction area of the vertical current is also consistent with our previous study^24^.

In order to investigate the effect of the trap site density of SiO_2_ film on the gate current flow, the vertical current behavior of MIM structures and TFT devices having 50 nm thick SiO_2_ insulator films with different film quality fabricated by thermal oxidation and PECVD (Plasma Enhanced Chemical Vapor Deposition) methods were compared. The MIM devices with 50 nm thick SiO_2_ grown by thermal oxidation at 1100 °C show little leakage current at the contact area of 0.2 mm^2^ and 0.79 mm^2^ (Fig. S4a,b), but the leakage current increases rapidly from the contact area of 3.14 mm^2^ (Fig. S4c,d). On the other hand, in the MIM devices with the 50 nm thick SiO_2_ deposited by PECVD, high leakage currents flow even at the contact area of 0.2 mm^2^ (Fig. S4e–h). The deviation of the leakage current flow due to the difference of the film quality is clearly displayed in the cumulative distribution graph in which the current value at 1 V is plotted (Fig. S5). The cumulative distribution graphs of leakage current demonstrate that the SiO_2_ thin-film deposited by PECVD permits much higher leakage current than the thermal oxidation even at the same junction size, which indicates that the SiO_2_ film by PECVD has much more trap density than the film formed by thermal oxidation. Based on the difference in quality of the SiO_2_ films, the *I*–*V* characteristics of the TFTs with 50 nm thick SiO_2_ gate insulator films grown by thermal oxidation and PECVD were compared. The area of the IGZO active layer in the two TFTs was equal to 1.5 mm^2^, and the structure of the TFTs was the same. The thermally oxidized 50 nm thick SiO_2_ insulator completely blocks the *I*_g_ (Fig. S6a), but a high uni-directional gate current flows through the 50 nm thick SiO_2_ film made by PECVD regardless of the same IGZO area (Fig. S6b); therefore, it can be rationally judged that the unusual gate current is due to the transport of electrons flowing through the trap sites in the insulator layer. On the other hand, in Fig. S4a,b, most devices with contact areas of 0.2 mm^2^ and 0.79 mm^2^ have little leakage current, while some devices allow a relatively high current above 10 nA. These results imply that the trap sites in the SiO_2_ thin-film are randomly distributed, thus some small areal regions have high trap density locally.

The TEM image of the thermally oxidized SiO_2_ thin-film interface reveals that the SiO_2_ film has a complete amorphous phase without any grains (Fig. S6c). This implies that Si and O are not in perfect stoichiometric binding state, and there are many defect-like trap sites in the film. It is a very difficult challenge to find the precise location of the defects in the amorphous thin-film through TEM analysis, but comparing the Si-O binding state using XPS analysis is a general method to estimate the amount of defects^31–33^. The XPS spectra of the Si 2 P levels for the thermally oxidized SiO_2_ and PECVD-deposited SiO_2_ films were compared, and a quartz glass, a SiO_2_ material of 99.95% high purity with almost no impurities, was used as a reference. The Si 2 P peak of the quartz glass is detected at a binding energy of 103.46 eV, which is consistent with preceding results of other studies^33^. On the other hand, the Si 2 P peak of the thermally grown SiO_2_ film is detected at lower binding energy of 103.35 eV, and that of the PECVD-deposited film is detected at 103.26 eV (Fig. S6d). The gradual shift of the Si 2 P peak toward lower binding energies means that SiO_2−X_ state, mainly Si^3+^, exists in both the thermally grown and the PECVD-deposited SiO_X_ compared to the quartz. The formation of Si^3+^ implies an increase in Si-rich bonds, such as dangling bonds or oxygen defects. This is clearly identified by each fitted peak: the peak of the quartz glass is mostly consisted of the Si^4+^, but the Si^3+^ peak hardly exists, and comparing the area of each peak, the Si^3+^ has a very small fraction of 0.01 compared to the Si^4+^ (Fig. S6e). On the other hand, the Si^3+^ peak occupies a larger portion in the thermally grown SiO_X_ film, and the area ratio of Si^3+^ is 0.18. The ratio of Si^3+^ peak in the PECVD-deposited SiO_X_ film is much larger and reaches 0.37 (Fig. S6f,g). Therefore, there are more defect states including dangling bonds and oxygen deficiencies in the thermally grown SiO_X_ film compared to the quartz, further, the PECVD-deposited SiO_X_ film has even more defect states than the thermally grown film. These analyzes agree well with the result that the 50 nm thick SiO_X_ film deposited by the PECVD allows higher leakage current than the same film grown by thermal oxidation (Figs S4 and S5). Meanwhile, the unusual leakage current phenomenon through the dielectric thin-film is not limited to the SiO_2_ material but is also confirmed in the Al_2_O_3_ thin-film, which is another typical dielectric material (Fig. S7). Furthermore, it is also confirmed that the work function difference between the bottom and top electrodes has little influence the vertical current through insulator films (Fig. S8). Consequently, it can be reasonable deduction that the electrical charge carriers are transmitted through the defect-like trap centers inherently present in SiO_X_ film. And it can be inferred that dangling bonds or atomic deficiencies in the fabricated amorphous SiO_X_ films are sites where electrical charge carriers can pass through.

The uni-directional *I*_g_–*V*_gs_ characteristics of the TFT are linearly plotted on log-log axis (Fig. S9), which means that the *I*_g_–*V*_gs_ relationship follows a power law. The slopes of the linearly fitted *I*_g_–*V*_gs_ characteristics are greater than 2.0 (Fig. S9b), which is well consistent with a theory of space charge-limited current (SCLC) *I*–*V* relationship, accounting for Ohmic type conductions through dielectric materials^34^. In the theory of the SCLC, when an electron density accumulated at the interface with an electrode contact is high enough to fill up the trap sites in a dielectric layer, the electrons becomes free to move in the dielectric^35^. Furthermore, it has been also reported that the SCLC is enhanced with increase of trap densities including bulk traps and surface traps in an insulator material^36,37^. Thus, the uni-directional gate current through the thick SiO_2_ insulator in the oxide TFT is due to the injected electrons from the IGZO active layer.

### Electrical charge states at the interface of the SiO_2_

**Figure 2:**
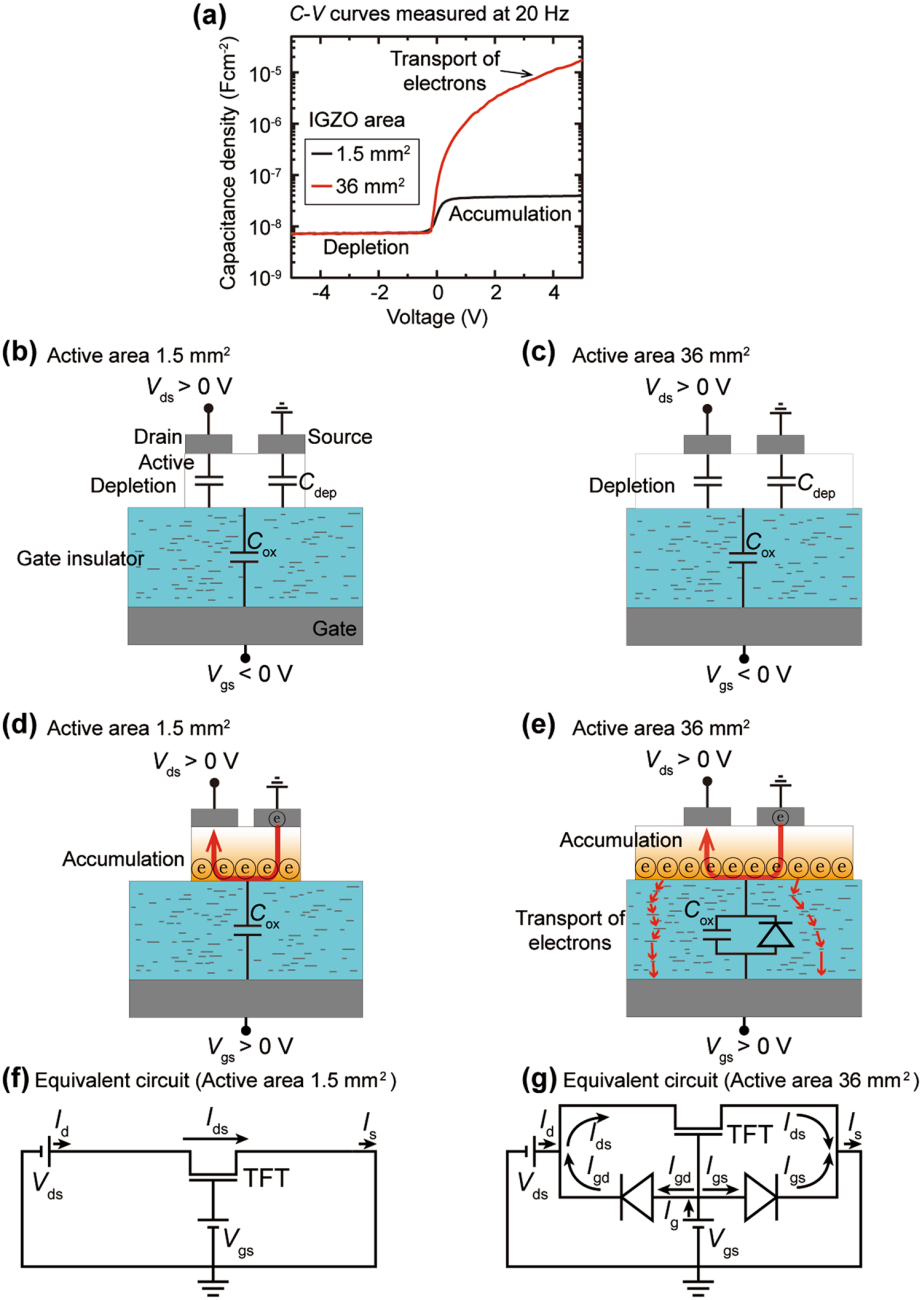
*C*-*V* curves and schematic images for the transport of electron in each TFT device. (**a**) *C*-*V* characteristics for the MOSCAP structures in each TFT device with different IGZO size of 1.5 mm^2^ and 36 mm^2^. (**b**,**c**) Surface depletion states and capacitances for turn-off condition of the TFTs with (**b**) 1.5 mm^2^ and (**c**) 36 mm^2^ IGZO area. (**d**,**e**) Surface accumulation states and electron transport for turn-on condition of the TFTs with (**d**) 1.5 mm^2^ and (**e**) 36 mm^2^ IGZO area. (**f**,**g**) Equivalent circuit of each TFT with (**f**) 1.5 mm^2^ and (**g**) 36 mm^2^ IGZO area.

The capacitance (*C*)–voltage (*V*) characteristics were measured at 20 Hz for the metal (P^++^ Si)-oxide (200-nm SiO_2_)-semiconductor (IGZO)-metal (source electrode) capacitor (MOSCAP) structures in each TFT with 1.5 mm^2^ and 36 mm^2^ IGZO active layers to compare the charge states at the SiO_2_ interface. The capacitances of two MOSCAPs measured in a negative *V*_gs_ range exhibit the same minimum values (Fig. 2a), which means that electrons, majority carriers in the IGZO layer, are fully depleted from the SiO_2_ interface. The fully depleted IGZO layer at the interface with the SiO_2_ creates an additional depletion capacitance (*C*_dep_) that is connected in series with the insulator capacitance (*C*_ox_). The *C*_dep_ values are the same regardless of the area of the IGZO layer; therefore, the depletion states of the two MOSCAPs are the same (Fig. 2b,c). However, in a positive *V*_gs_ region, the *C* values of two MOSCAPs exhibit exactly the opposite behavior. The *C* value for the MOSCAP having 1.5 mm^2^ area IGZO layer is maintained at 3.9 × 10^−8^ Fcm^−2^, which indicates that the electrons in the IGZO layer are accumulated at the interface and the total capacitance value corresponds to *C*_ox_ of 200 nm thick SiO_2_ (Fig. 2d). On the other hand, the *C* of the MOSCAP with 36 mm^2^ area IGZO film increases steadily with the positive *V*_gs_ and reaches 1.7 × 10^−5^ Fcm^−2^ at 5 V. The increasing *C* behavior demonstrates that the electrons flow steadily from the accumulated IGZO interface into the bottom electrode through the SiO_2_ film. The SiO_2_ layer serves not only as a gate dielectric in the TFT structure, but also as an electron transport layer such as a diode (Fig. 2e). The different characteristics are also verified in the conductance-voltage and series resistance-voltage characteristics for the MOSCAP devices (Fig. S10). Consequently, there are no leakage current in the TFT having 1.5 mm^2^ area IGZO layer, and the equivalent circuit is depicted as shown in Fig. 2f, and the only current path in the TFT is the channel layer consisting of the 20-nm IGZO. Conversely, in the TFT having a large active area (36 mm^2^), high current flows not only between the drain and source electrodes (*I*_ds_), but also between the drain and gate electrodes (*I*_gd_) and between the source and gate electrodes (*I*_gs_). The equivalent circuit for the TFT with the uni-directional gate current can be described as including the vertical current paths like a diode (Fig. 2g). As can be expected from the equivalent circuit, the *I*_d_ begins to decrease from the operating region where the *I*_gd_ injected into the drain electrode is larger than the *I*_ds_ flowing out from the drain electrode, therefore, the *I*_d_ curve in log axis seems to be distorted in Fig. 1e.

### Influence of the structural parameters of TFT on the gate current

**Figure 3:**
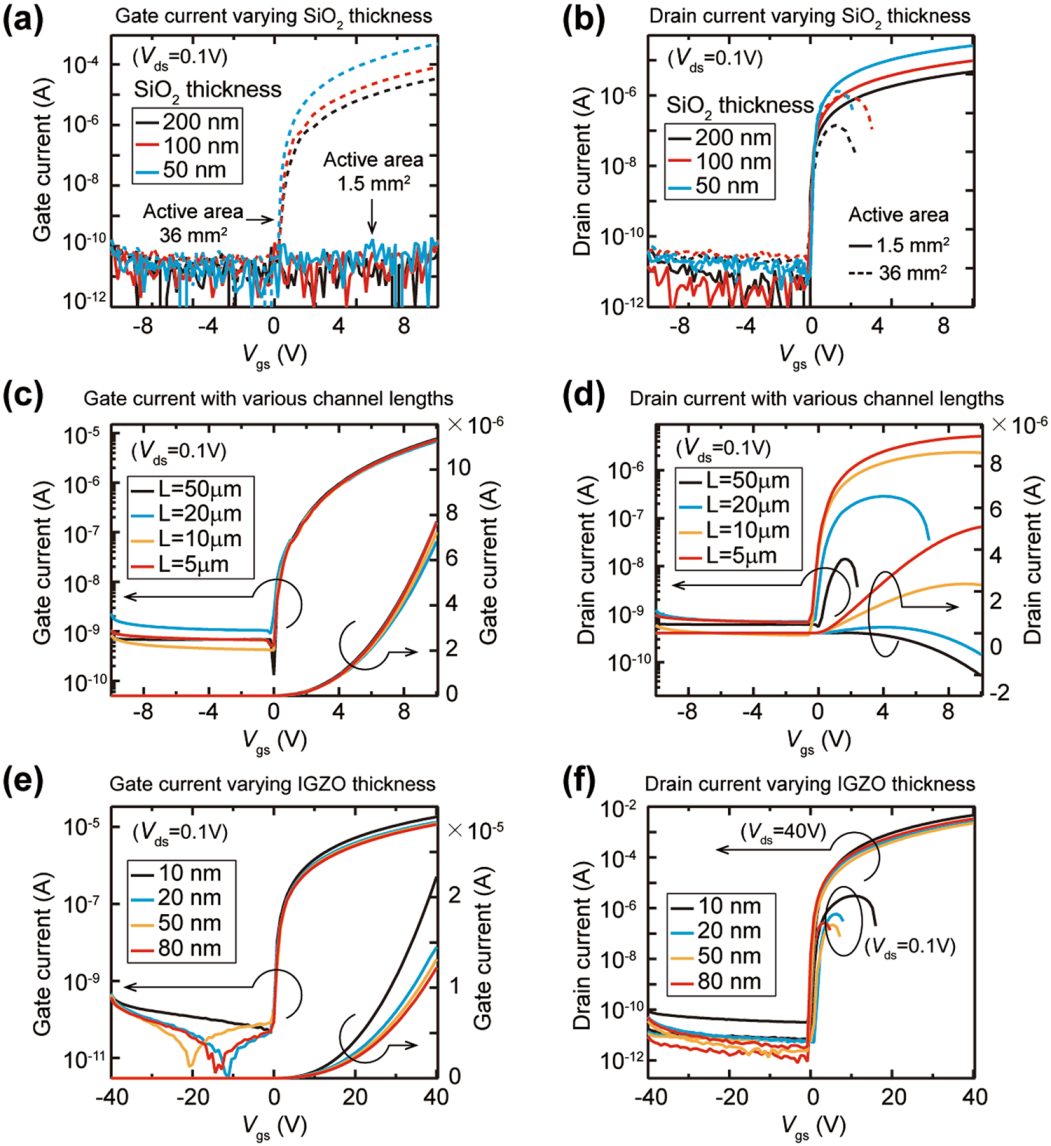
Gate and drain current behavior with variation of structural parameters of the TFT. (**a**,**b**) The electrical characteristics measured at *V*_ds_ of 0.1 V for the TFTs with 50-nm, 100-nm, and 200-nm SiO_2_ gate insulator layers. (**a**) Gate current and (**b**) drain current behavior for the TFTs with 1.5 mm^2^ and 36 mm^2^ IGZO area. (**c**,**d**) The gate and drain current behavior with various channel length in the TFTs with 36 mm^2^ IGZO active layer. (**c**) Gate current values of the TFTs are equivalent for all channel lengths. (**d**) As a channel length becomes longer, the specific *V*_gs_ value at which the drain current starts to decrease is gradually reduced. (**e**,**f**) The effect of the thickness of IGZO active layer on the drain current in the TFTs with 36 mm^2^ IGZO active layer. (**e**) Gate current values of the TFTs are equivalent for all thickness of the IGZO layer. (**f**) The effect of the gate current on the drain current is similar regardless of the IGZO layer thickness.

The uni-directional gate current in the oxide TFT is due to the transport of electrons between the gate electrode and the drain and source electrodes. Therefore, the gate current is influenced by the magnitude of electric fields in the oxide TFT and is controlled by the structural parameters of the oxide TFT including the thickness of gate dielectric, the channel length, and the thickness of active layer. The gate current depends on the vertical electric field applied to the SiO_2_ gate dielectric, and the vertical electric field varies with the thickness of the SiO_2_ gate dielectric. Because the vertical electric field between the gate electrode and the source electrode increases as the thickness of the SiO_2_ decreases, the gate current through the SiO_2_ gate dielectric increases steadily as the thickness of the gate dielectric decreases from 200 nm to 50 nm (Fig. 3a). As confirmed in the Figs S1–S3, the uni-directional gate current flows stably in the oxide TFT with a large area (36 mm^2^) IGZO active layer, but there is absence of the gate current in the oxide TFT with a small area (1.5 mm^2^) IGZO active layer. Therefore, the drain current proportionally increases with the *V*_gs_ in a TFT having an active layer of a small area, whereas in a TFT with a large area active layer, the drain current starts to decrease from a low *V*_gs_ due to the influence of the gate current (Fig. 3b). In order to control the transverse electric field between the drain electrode and the source electrode, the channel length varied from 5 μm to 50 μm. The tested TFTs have a IGZO active layer of 36 mm^2^ area and a SiO_2_ dielectric layer of 200 nm thickness. The vertical electric field through the SiO_2_ layer is invariant regardless of the channel length, therefore, the uni-directional gate currents are equivalent for all channel legnths (Fig. 3c). On the other hand, as the channel length increases from 5 μm to 50 μm, the transverse electric field decreases and the drain current through the channel layer decreases; thus, as the channel length increases, the gate current value becomes larger than the drain current value, and the drain current is seriously affected by the gate current and decreases at a smaller *V*_gs_ (Fig. 3d). We also investigated the effect of IGZO thickness on the gate current flow in the oxide TFT. The uni-directional gate current is due to the accumulated electrons at the interface with the SiO_2_ insulator, and the accumulated surface electron state is similar for all thickness of IGZO layer; thus, the gate current values are similar regardless of the IGZO layer thickness from 10 nm to 80 nm, and the thickness of IGZO active layer has little effect on the change in the gate current (Fig. 3e,f).

### Demonstration of gate current path as an ESD diode component

**Figure 4:**
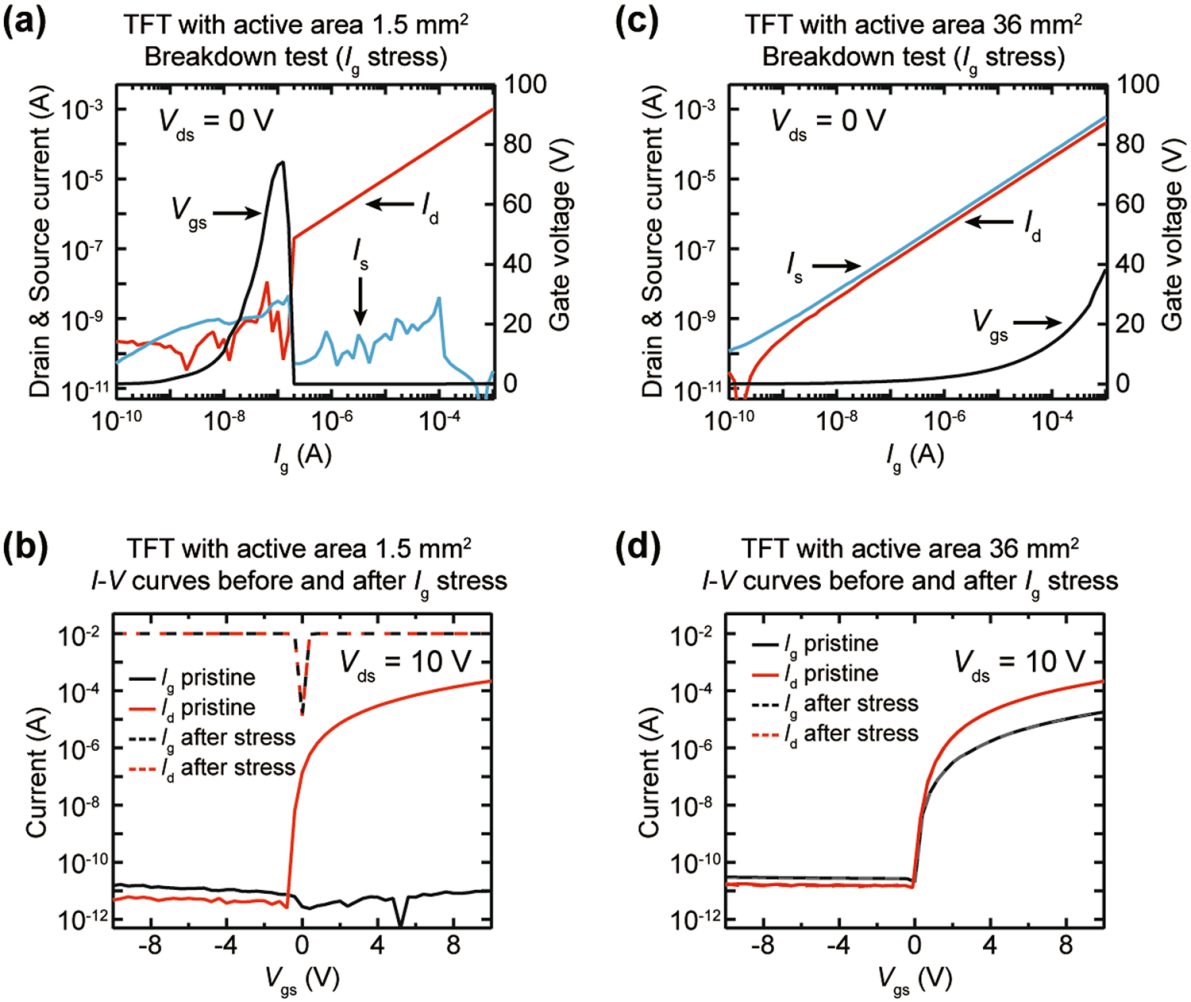
Demonstrating applicability of the unusual gate current paths as an electrostatic protection circuit. (**a**,**b**) Results of *I*_g_ stress test for the TFT with 1.5 mm^2^ active layer. (**a**) Dielectric breakdown occurs between the gate and drain electrodes due to vertical electric field as *I*_g_ increases. (**b**) An electrical shorting occurs between the gate and drain electrodes due to the dielectric breakdown by *I*_g_ stress. (**c**,**d**) Results of *I*_g_ stress test for the TFT with 36 mm^2^ active layer. (**c**) The gate current flows to the drain and source electrodes at the same time without a dielectric breakdown. (**d**) The TFT output values exhibit the same value before and after *I*_g_ stress.

Although the high gate current path increases the power consumption of the TFT, it has a huge potential as an electrostatic discharging (ESD) diode of a driving circuit. Therefore, we demonstrated that the stable gate current path through the thick SiO_2_ layer prevents a dielectric breakdown of SiO_2_ film from an excessive static charge coming in the gate direction. The oxide TFTs having 100 nm thick SiO_2_ gate dielectric were investigated. The *I*_d_, *I*_s_, and *V*_g_ outputs in response to the applied *I*_g_ were measured at the drain, source, and gate electrodes, respectively, while the *I*_g_ of 10^−10^ A to 10^−3^ A was applied to the gate electrode. The drain voltage was maintained at 0 V. In the TFT with a small area (1.5 mm^2^) IGZO layer, the applied *I*_g_ causes a sharp increase in *V*_g_ because electrical charge carriers cannot move vertically; thus, *V*_g_ reaches about 80 V at the *I*_g_ of 10^−7^ A (Fig. 4a). Then, a sudden voltage drop in *V*_g_ occurs near the *I*_g_ of 2 × 10^−7^ A, and the *I*_d_ begins to increase linearly with *I*_g_, while *I*_s_ maintains the low current states. The result indicates that an excessive vertical electric field between the gate and drain electrodes, which is induced by the *I*_g_, leads to a sudden dielectric breakdown of the SiO_2_ film, and most of the *I*_g_ is leaked to the drain electrode. With the dielectric breakdown, the TFT characteristics before and after the *I*_g_ stress drastically changed: both of the *I*_g_ and *I*_d_ exhibit an Ohmic-like conduction due to electrical shorting (Fig. 4b). In contrast, in the TFT with a large area (36 mm^2^) IGZO layer, the *I*_d_ and *I*_s_ increase linearly in proportion to the *I*_g_, which indicates that the input *I*_g_ escapes simultaneously to the drain and source electrodes. The *I*_g_-induced potential *V*_g_ is lower than that of the TFT with a small area IGZO and reaches about 40 V at the *I*_g_ of 10^−3^A (Fig. 4c). Thus, the path of the gate current through the SiO_2_ insulator functions as a diode for discharging the electrostatic overcurrent, and the TFT is protected from an electric breakdown and maintains its output capability (Fig. 4d).

## Discussion

In summary, it is studied that a high leakage current flows very reliably through a thick insulator film between electrodes without any dielectric breakdown by controlling the trap site density in the insulator layer. It is reasonably verified that the origin of the high vertical current is due to the transport of injected electrons from the electrode contact, and the electrons are transported through trap sites inherently present in the insulator layer. The vertical current can be controlled uni-directionally through control of the semiconductor charge states at the dielectric interface in the MIS structure using the oxide semiconductor layer. The uni-directional leakage current in oxide TFTs interferes with the electrical performance of the oxide TFTs, thus, the oxide semiconductor active layer should be patterned to prevent the interference of the gate current. Although the unconventional gate current increases the power consumption of the TFT, the uni-directional gate current path can efficiently discharge the excessive charges and protect the TFT from the permanent breakdown when an excessive electrostatic charge is applied in the gate direction. Our study of the origins of abnormal gate currents provides a clear interpretation and simple solution for unknown factor in oxide TFT structures and oxide-insulator junctions. Moreover, our approach to reliably controlling vertical current through thick insulators will stimulate a new perspective interest in oxide hetero-interfaces or oxide electronics research.

## Methods

### Deposition of IGZO oxide semiconductor

The deposition of the IGZO oxide semiconductor layer was performed by RF magnetron sputtering system using indium gallium zinc oxide (In:Ga:Zn:O = 1:1:1:4 at%) target under 10^−6^ Torr in room temperature. High purity Ar gas was the only reactive sputtering gas. After deposition process, IGZO film was annealed at 350 °C for 90 seconds in air through a rapid thermal annealing (RTA) method.

### Deposition of dielectric films

The silicon dioxide (SiO_2_) was oxidized onto P^++^ Si wafer at 1100 °C by a conventional thermal oxidation process, and deposited by PECVD using SiH_4_ and N_2_O gases. The aluminum oxide (Al_2_O_3_) dielectric films were deposited by atomic layer deposition (ALD) technique and solution process which is to dissolve 0.2 M aluminum nitrate nonahydrate in 10 mL of the 18.2 MΩ deionized water at 25 °C.

### Fabrication of thin-film transistors (TFTs)

Highly Boron-doped p-type semiconductor substrate with resistivity of 0.005 Ωcm. The SiO_2_ dielectric films were formed onto P^++^ Si wafer, and the oxide semiconductor films were patterned as an active layer, then the source and drain electrodes of 100 nm thickness were grown by a thermal evaporation tool under 10^−6^ Torr in room temperature. The width and length of the channel in TFTs are 1000 μm and 50 μm, and the narrower channel lengths of 5, 10, 20, and 50 μm in the Fig. 4 were patterned by a conventional photolithography and wet etching process. Most of the source and drain electrodes and all of the oxide semiconductor films were patterned by metal shadow masks in order to avoid unexpected side-contacts with gate electrode.

### Characterization of the devices

The current - voltage and capacitance - voltage characteristics for all devices were measured using the Agilent 4155B semiconductor parameter analyzer and the Agilent 4284 A precision LCR meter in a dark.

## Electronic supplementary material

Supplementary information

## Data Availability

The datasets generated during and/or analyzed during the current study are available from the corresponding author on reasonable request.
