# Supplementary material for: Effects of Unusual Gate Current on the Electrical Properties of Oxide Thin-Film Transistors
Source: Sci Rep. 2018 Sep 17;8:13905. doi: 10.1038/s41598-018-32233-4 (PMC6141615; doi:10.1038/s41598-018-32233-4)
Supplement: Supplementary file 1 — Supplementary information [file 41598_2018_32233_MOESM1_ESM.docx]

**Supplementary Information**

**Effects of Unusual Gate Current on the Electrical Properties of Oxide Thin-Film Transistors**

**Jinwon Lee^1^, Keon-Hee Lim^1^, and Youn Sang Kim^1,2*^**

^1^Program in Nano Science and Technology, Graduate School of Convergence Science and Technology, Seoul National University, 1 Gwanak-ro, Gwanak-gu, Seoul 08826, Republic of Korea

^2^Advanced Institute of Convergence Technology, 145 Gwanggyo-ro, Yeongtong-gu, Suwon 16229, Republic of Korea

^*^Corresponding author; Youn Sang Kim (E-mail: [younskim@snu.ac.kr](mailto:younskim@snu.ac.kr), tel: 82-31-888-9131)

**Note 1. Comparison of leakage current through 200-nm SiO_2_ insulating layer with changes in electrode area**

In order to investigate the leakage current behavior through the SiO_2_ insulator layer with a 200 nm thickness, the MIM structure consisted of P^++^ Si / thermally oxidized 200-nm SiO_2_ / 100-nm Al was fabricated. The top Al electrode has a circle shape in a diameter from 0.5 mm to 6 mm. 40 devices for each MIM device with different Al electrode area were investigated for a reliable comparison (Figure S1). Furthermore, the leakage current characteristic was also explored in the MIS structure composed of of P^++^ Si / thermally oxidized 200-nm SiO_2_ / 20-nm IGZO, which is the same structure as in the bottom gate oxide TFT. In the MIS devices, the leakage current flows in only one direction, which is from the bottom electrode toward top IGZO layer; in other words, electrons are transported only from the IGZO layer to the P^++^ Si electrode (Figure S2). As the top electrode area increases in the MIM and MIS devices, the probability of the leakage current through the 200-nm SiO_2_ layer increases. The probability is summarized in the cumulative distribution graphs of the leakage current values measured at 1 V in the MIM devices and 10 V in the MIS devices (Figure S3). The cumulative distribution graphs for the MIM and MIS devices show that very negligible leakage current flows uniformly in structures with a top electrode of 0.2 mm^2^ area. The probability of the leakage current steadily increases with the area of the top electrode and the distribution of the leakage current value in the device having the top electrode of 36 mm^2^ becomes highly uniform to a level higher than 10^-6^ A.


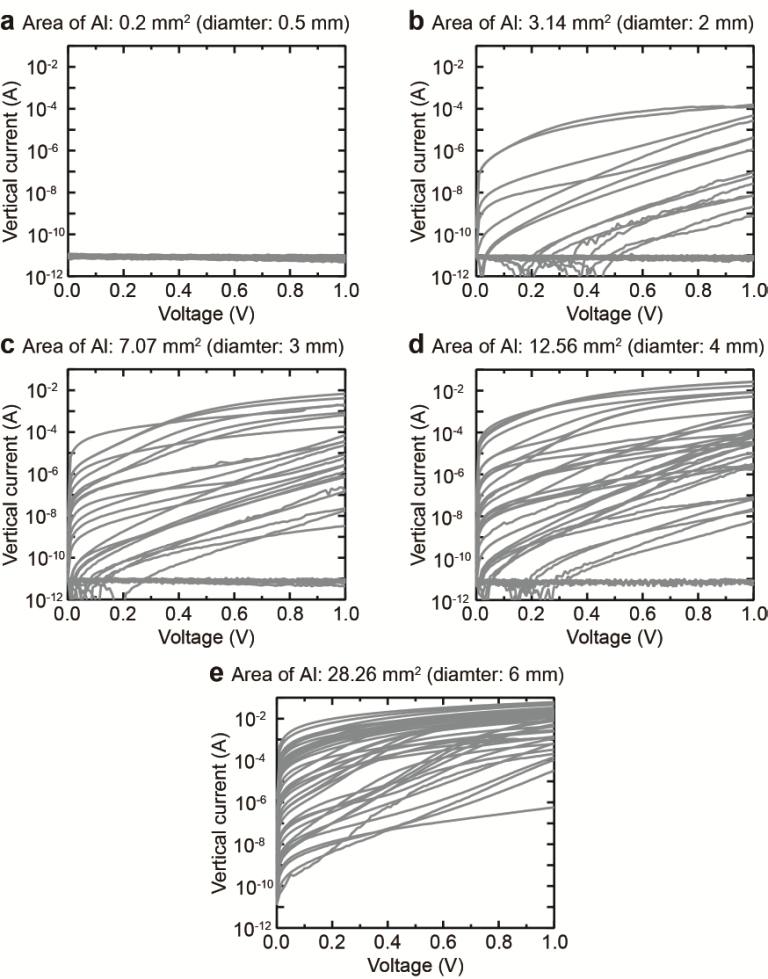


**Figure S1. Leakage current flowing in metal / insulator / metal (P^++^ Si / 200-nm SiO_2_ / 100-nm Al) structures with various Al electrode area from 0.2 mm^2^ to 28.26 mm^2^.** The Al electrodes have circular shapes in a diameter (a) 0.5 mm, (b) 2 mm, (c) 3 mm, (d) 4 mm, and (e) 6 mm. 40 devices were measured for each size.


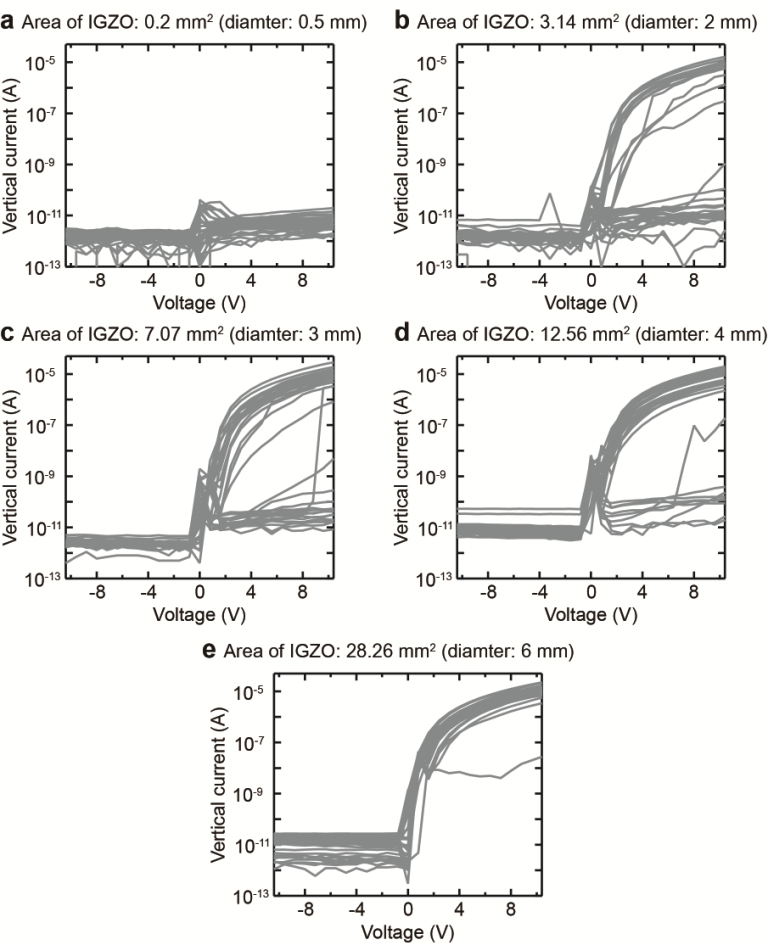


**Figure S2. Leakage current flowing in metal / insulator / oxide semiconductor (P^++^ Si / 200-nm SiO_2_ / 20-nm IGZO) structures with various IGZO area from 0.2 mm^2^ to 28.26 mm^2^.** The IGZO layers have circular shapes in a diameter (a) 0.5 mm, (b) 2 mm, (c) 3 mm, (d) 4 mm, and (e) 6 mm. 40 devices were measured for each size.


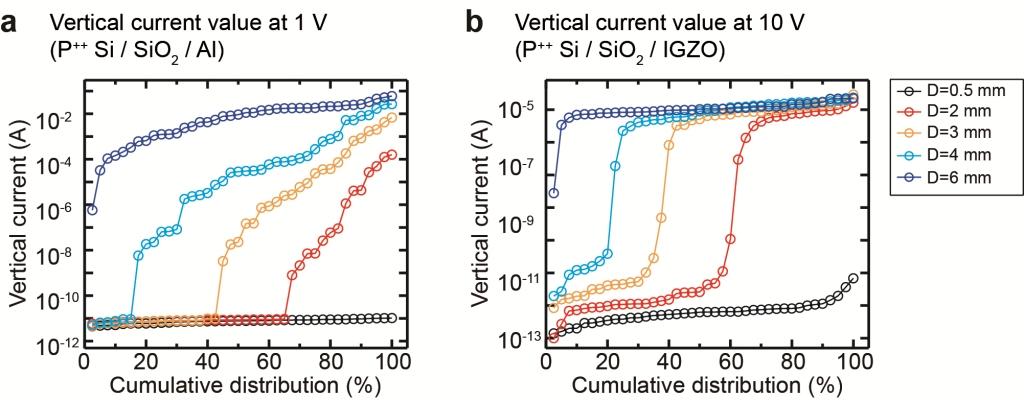


**Figure S3. Cumulative distribution of the leakage current values for the MIM and MIS devices.** (a) Cumulative distribution of the leakage current measured at 1 V for the MIM (P^++^ Si / 200-nm SiO_2_ / 100-nm Al) devices with various junction sizes. (b) Cumulative distribution of the leakage current measured at 10 V for the MIS (P^++^ Si / 200-nm SiO_2_ / 20-nm IGZO) devices with various junction sizes.


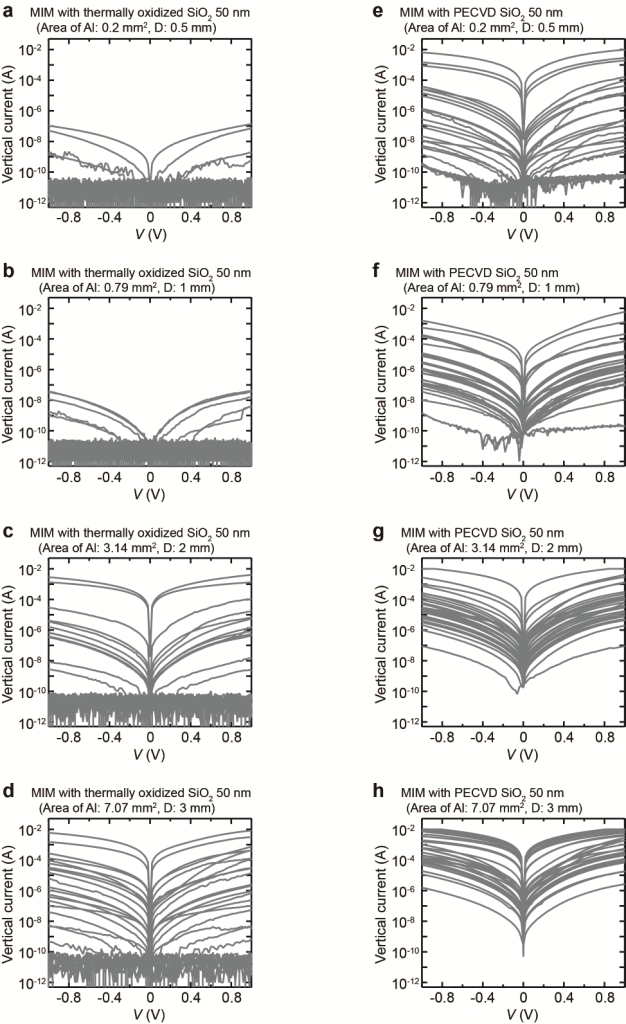


**Figure S4. Comparison of vertical current flowing by SiO_2_ deposition method in metal / insulator / metal (P^++^ Si / 50-nm SiO_2_ / 100-nm Al) structures with various Al electrode area from 0.2 mm^2^ to 7 mm^2^.** The SiO_2_ films were formed by (a-d) thermal oxidation and (e-h) PECVD method. The Al electrodes have circular shapes in a diameter (a,e) 0.5 mm, (b,f) 1 mm, (c,g) 2 mm, and (d,h) 3 mm. 30 devices were measured for each size.


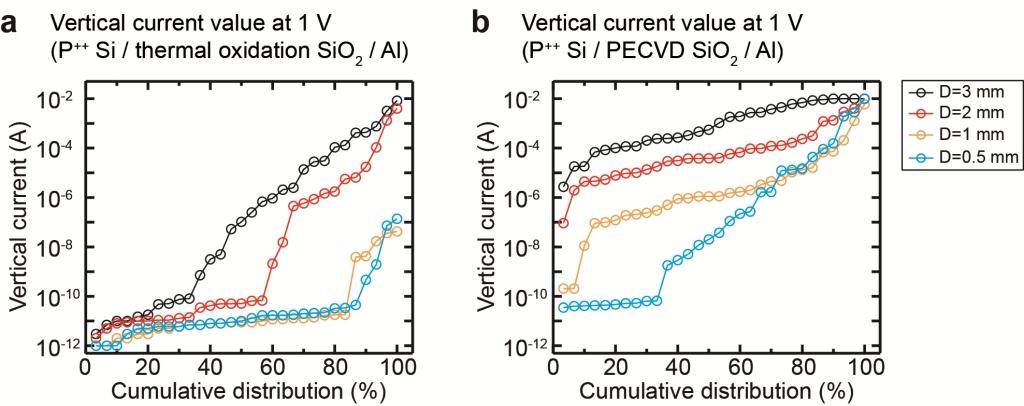


**Figure S5. Cumulative distribution of vertical current values measured at 1 V of MIM devices with 50-nm SiO_2_ layers of different film quality.** (a,b) Cumulative distribution of the current measured at 1 V for the MIM devices with (a) thermally oxidized and (b) PECVD deposited 50-nm SiO_2_ insulators varying junction sizes.


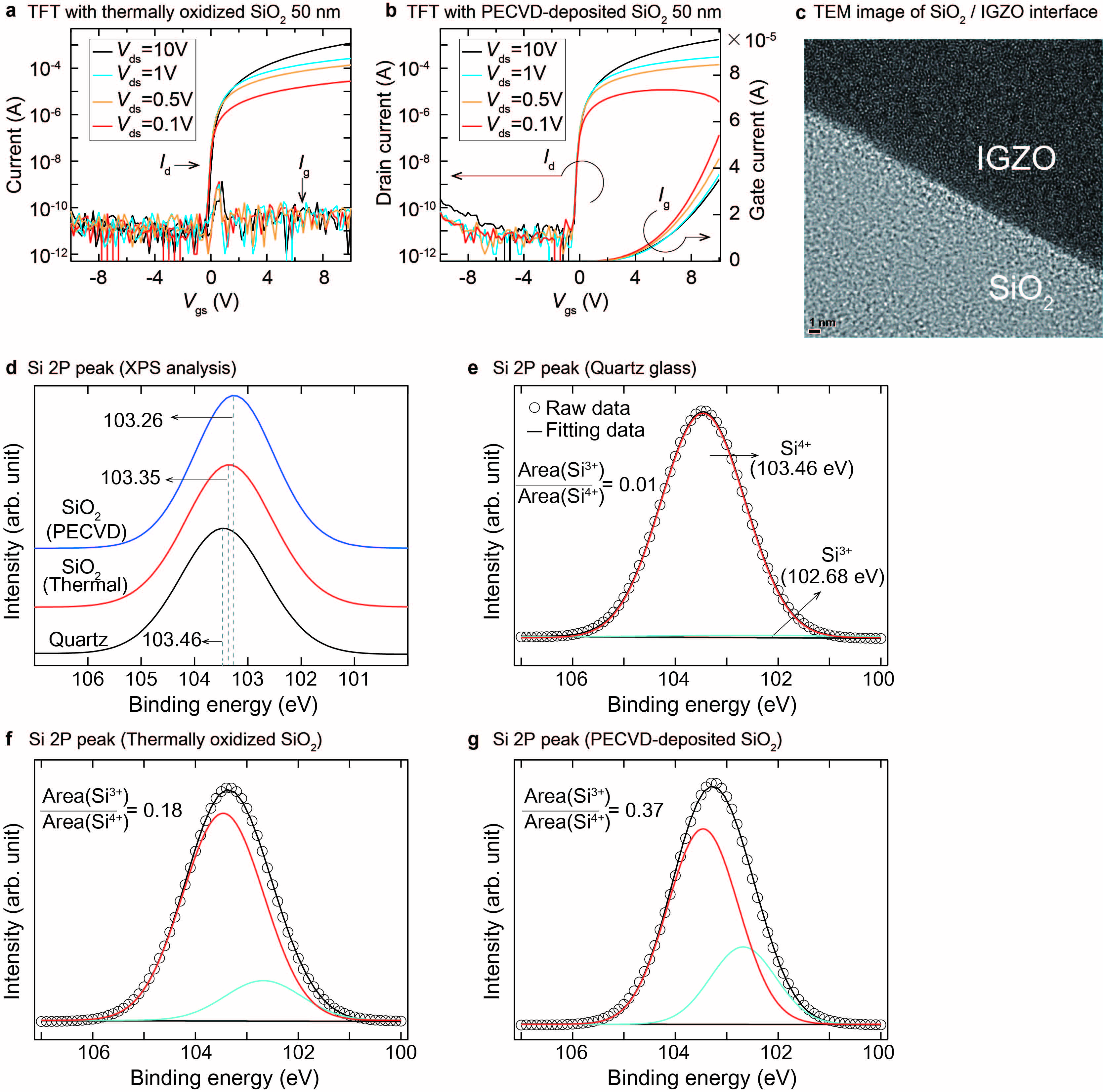


**Figure S6. Comparison of leakage current through SiO_2_ films used as gate dielectrics in TFTs and XPS analysis.** The *I*-*V* characteristics for the TFTs with SiO_2_ gate dielectric layers of 50 nm thickness which are formed by (a) thermal oxidation and (b) PECVD process. (c) TEM image of the SiO_2_ / IGZO interface. (d) XPS spectra of Si 2P for (e) a quartz glass, (f) the thermally oxidized SiO_2_, and (g) PECVD-deposited SiO_2_.

**Note 2. Investigation the vertical current phenomena in the MIM devices with AlO_X_ dielectric films**

In order to verify whether the abnormal vertical current flow through the dielectric films is limited to the SiO_2_ material, we investigated a representative dielectric material, AlO_X_. The AlO_X_ thin-films were deposited by ALD vacuum deposition method and solution coating method in order to confirm the influence of the growth process of dielectric thin films. The thickness of Al_2_O_3_ films is equal to 20 nm. The 100 nm thick Al film was used as top electrode. The Al top electrode size with circle shape was compared from 0.5 mm diameter to 2 mm diameter. We measured and compared 15 devices for each condition. In the MIM devices with the smallest Al contact area of 0.5 mm diameter, the vertical current hardly flows in the both AlO_X_ dielectric films formed by ALD method and the solution process (Figure S7a and d). However, as the contact area increases, the bi-directional vertical current begins to flow and the probability of high current flow increases significantly in both AlO_X_ films (Figure S7b, c, e and f). The cumulative distributions clearly show that the tendency of the vertical current flowing in the MIM device is equal even if the AlO_X_ film is deposited in different process (Figure S7g and h). Further, the MIS devices using AlO_X_ dielectric films, which are deposited by ALD and solution process, exhibit the same *I*-*V* characteristic: the vertical current flows in only one direction from the bottom electrode to the top electrode (Figure S7i). Therefore, it is confirmed that the abnormal vertical current flowing through the dielectric film is a universal electric phenomenon that can occur in the MIM or MIS structure. It is also confirmed that the film quality of the dielectric film itself or the size of the electrode in contact with the dielectric film is closely related to the vertical current regardless of the insulator material or the process method.

**
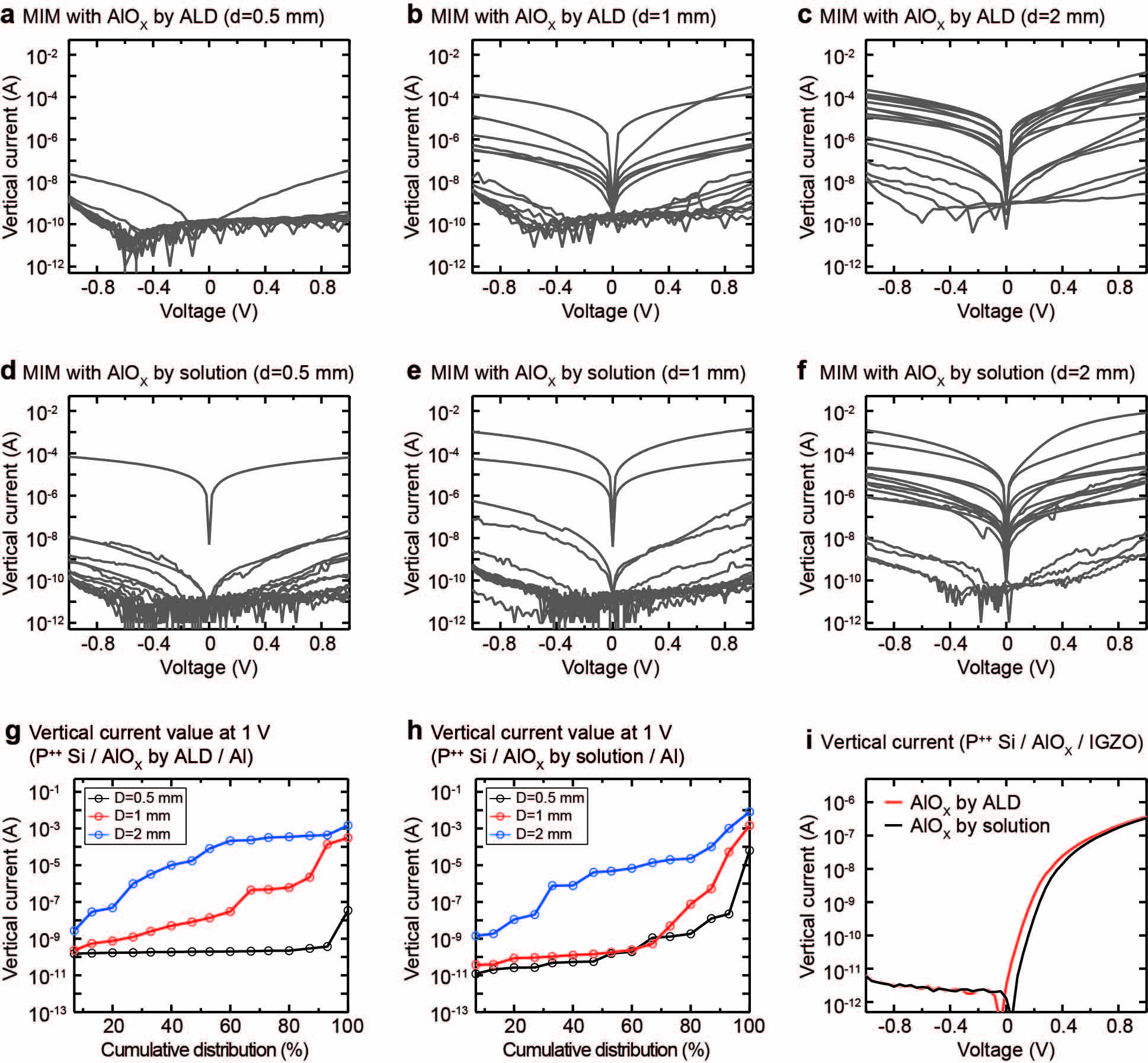
**

**Figure S7. Comparison of leakage current through AlO_X_ films by grown ALD method and solution process in the MIM (P^++^ Si / 20-nm AlO_X_ / 100-nm Al) structures.** The AlO_X_ films were formed by (a-c) ALD and (d-f) solution process. The Al electrodes have circular shapes in a diameter (a,d) 0.5 mm, (b,e) 1 mm, and (c,f) 2 mm, and 15 devices were measured for each size. The cumulative distribution graphs for the leakage current value measured at 1 V for the MIM structures with (g) ALD-deposited AlO_X_ and (h) solution-coated AlO_X_. (i) The *I*-*V* curves for the MIS devices using AlO_X_ dielectric films.

**Note 3. Influence of the work function difference on the unusual vertical current behavior**

The influence of the work function difference on the unusual vertical current behavior is investigated. First, we compared the *I*-*V* behavior of P^++^ Si / 200-nm SiO_2_ with Al and Au top electrodes, respectively. As is well known, there is a work function difference of about 0.5 eV or more between Al and Au metals. The *I*-*V* characteristics for the devices using Al top electrodes are drawn in Figure S1 and S3. The results demonstrate that the unusual leakage current through the 200 nm thick SiO_2_ bi-directionally flows in the MIM devices with Al contact and the current increases as the Al contact size increases. We investigated the devices consisted of P^++^ Si / 200-nm SiO_2_ / Au top electrode in the same condition. The unusual vertical current through the SiO_2_ film flows also bi-directionally in the devices using Au top electrode, and the current value also increases as the Au contact size increases from 1 mm to 3 mm diameters (Figure S8a-d). Therefore, it is confirmed that the work function difference between the top and bottom electrodes has little influence on the unusual leakage current behavior in our MIM devices. And it can be explained that the electrical charge carriers is transported through the trap sites present within the forbidden bandgap of the dielectric film, thus, the work function or contact barrier height has little effect on the current flow. The result confirms that the properties of the dielectric film and an electrode contact area are crucial factor to control the unusual vertical current.

On the other hand, the unconventional bi-directional current flowing through the dielectric films is controlled by the uni-directional current when the top electrode is changed to an oxide semiconductor. That is, the vertical current flowing in both directions in the MIM structure flows in only one direction due to the interface charge state control of the oxide semiconductor electrode in the MIS devices. We also have investigated the influence of work function difference on the uni-directional current in the MIS device. In the bottom electrode / 200 nm thick SiO_2_ / IGZO electrode structure, the bottom electrode was changed into highly doped P-type Si, highly doped N-type Si, and ITO with different work functions, respectively, and the *I*-*V* behavior was compared. In particular, there is a clear work function difference of about 1 eV between highly doped P-type and N-type Si, so it is appropriate to confirm the work function effect. All of the measured *I*-*V* curves for the devices with each bottom electrode are almost the same, which shows that the injection of charge carriers into the dielectric insulator layer is independent of the work function level of the bottom electrode (Figure S8e). Therefore, the injection and transport of the carrier through the insulating film is not affected by the height of the energy barrier at each interface, confirming that the charge carriers are transferred through inherently distributed trap centers of the insulator layers.

**
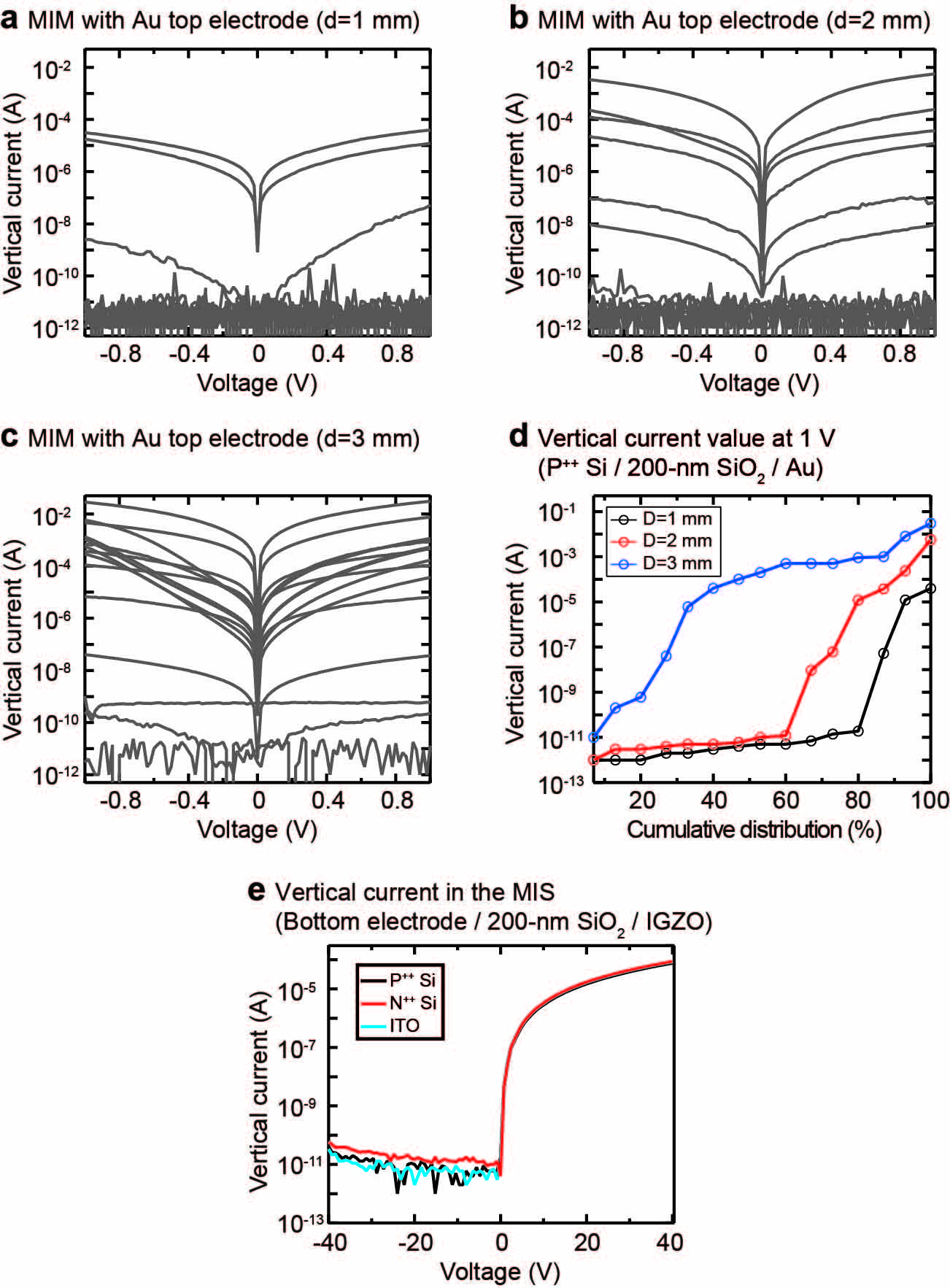
**

**Figure S8. Investigation for the influence of the work function difference between two electrodes.** (a-d) The vertical current behavior in the MIM devices (P^++^ Si / 200-nm SiO_2_ / 100-nm Au) with various Au top electrode size. The Au electrodes have circular shapes in a diameter (a) 1 mm, (b) 2 mm, and (c) 3 mm, and 15 devices were measured for each size. (e) The vertical current behavior in the MIS devices (bottom electrode / 200-nm SiO_2_ / 20-nm IGZO). The bottom electrode was compared among P^++^ Si, N^++^ Si, and ITO having different work functions.

**Note 4. Conduction mechanism of electrons flowing through a thick SiO_2_ layer**

In conduction theory in dielectrics, the electrical charge carriers are transported through a dielectric film by a number of conduction mechanisms.^1,2^ Among the well-established conduction mechanisms, an ohmic conduction and a space charge-limited conduction mechanisms are dependent mainly on the properties of the dielectric itself. In ohmic conduction, electrical charge carriers including electrons and holes are transported in mobile bands, conduction band and valence band. However, the high energy barrier heights between the dielectric and the contact metal prevent the charge carriers from being implanted into the mobile bands. Nevertheless, a small number of carriers that can be excited by an external thermal energy are present in the dielectrics, which contribute to the ohmic conduction. Although the ohmic conduction partially contributes to other dielectric conductions, but in a resistive switching device that has been actively studied recently, conduction in a low resistance state, in which current flows through a conductive filament, has been found to be an ohmic conduction.^3,4^ In the ohmic conduction, the electrical current through a dielectric exhibits a linear relation to the electric potential (i.e., the electric field) like equation (1), thus, the slope of the linearly fitted *I*-*V* curve is close to 1.0.

$J=\sigma E$, (1)

where *J* is an electrical current density value, *E* is the constant electric field in a dielectric, and *σ* is electrical conductivity.

In the case of space charge-limited conduction, electrons are injected into the dielectrics from the contact metal with negligible resistance. When the injected electrons fill up the trap sites in the dielectric, a space charge starts to build up in the dielectric; the injected electrons then move freely within the dielectric. In the condition of very strong injection of electrons, all trap sites are filled up and the conduction becomes to be limited by the space charge density. Furthermore, the current density (*J*)-electric potential (*V*) characteristic of the space charge-limited current follows the Child’s raw, and the space charge-limited current can be expressed as:

$J=\frac{9}{8}\mu_{e}\varepsilon_{0}\varepsilon_{ox}\frac{V^{2}}{t_{ox}^{3}}$, (2)

where *μ*_e_ is the mobility of electron, *ε*_0_ is the permittivity of the free space, *ε*_ox_ is the relative permittivity of the dielectric oxide, and *t*_ox_ is the thickness of the dielectric. The space charge-limited current is proportional to *V*^2^; therefore, the *I*-*V* characteristics plotted on the log-log axis should have a linear relationship of slope of around 2.0. In our experiments, the slopes of the linearly fitted gate current – gate voltage curves are around 2.3 (Figure S9b), which means the unusual gate current in the oxide TFT depends on the space charge-limited conduction. Consequently, the origin of the uni-directional gate current in the oxide TFT can be judged as electrons passing through the trap site present in the SiO_2_, and the conduction of electron is governed by the space charge-limited conduction mechanism.


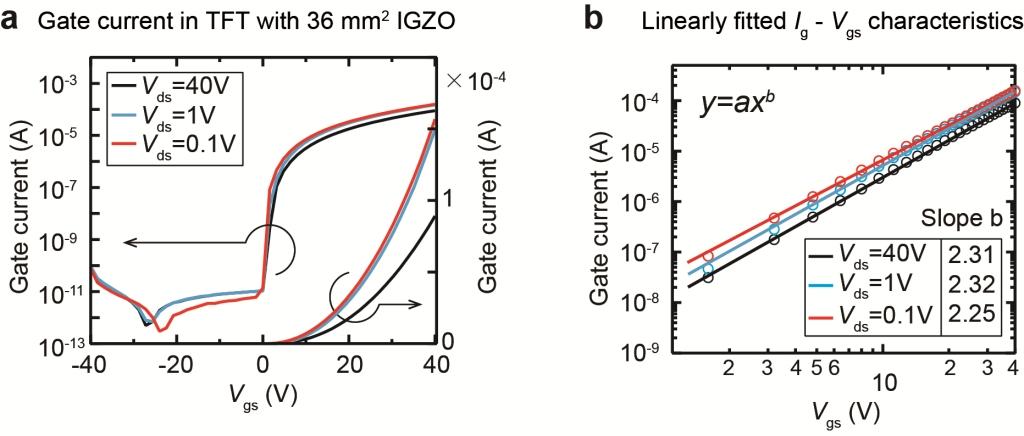


**Figure S9. Conduction mechanism of electrical charge carriers estimated by the *I*_g_ – *V*_gs_.** (a) Gate current flows in the TFT with 36 mm^2^ IGZO active layer. (b) The linearly plotted gate current – gate voltage curves in log-log axes.


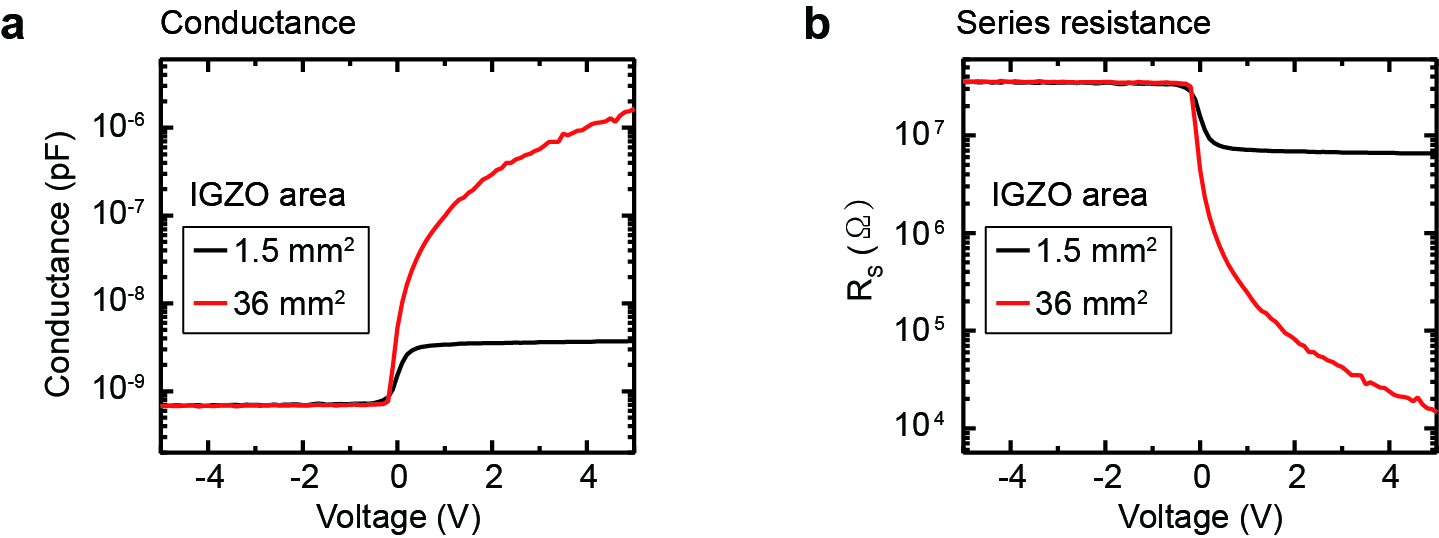


**Figure S10. (a) Conductance-voltage characteristics and (b) series resistance-voltage characteristics of** the MOSCAP structures in each TFT device with different IGZO size of 1.5 mm^2^ and 36 mm^2^.

**Note 5. Series resistance of the MOSCAP devices with different IGZO oxide semiconductor area**

In order to verify more correctly the electron transport of the interfacial layers of the MOSCAP devices with different IGZO area, the conductance-voltage relation was also investigated. Also, series resistances in the MOSCAP devices were calculated and compared. In MIS structures, the capacitance-voltage, conductance-voltage, and series resistance-voltage relations supply more correct and detail results about charge carrier transporting in the interfaces between semiconductor and insulator layers.^5^ Conductance values for the MOSCAP with 1.5 mm^2^ IGZO semiconductor layer are flat in the accumulation region (V > 0). However, conductance values for the MOSCAP with 36 mm^2^ IGZO layer increase with voltage in the accumulation region (Figure S10a). The difference is the same result to the *C*-*V* characteristics in Figure 2a. The series resistance (*R*_s_) of the MOSCAP structures also exhibits completely different patterns. The series resistance was extracted and calculated by following method that has been proposed by several researchers.^5^

$R_{s}=\frac{G}{G^{2}+(2\pi fC)^{2}}$, (2)

where *C*, *G*, and *f* are the measured capacitance, conductance values, and measuring frequency, respectively, in the accumulation regime. The *R*_s_ of the MOSCAP with 1.5 mm^2^ IGZO film has negligible change in the accumulation region, but the *R*_s_ of the MOSCAP with 36 mm^2^ IGZO layer decreases steadily with voltage (Figure S10b).

Eventually, the capacitance, conductance, and series resistance characteristics of the MOSCAP structure with a large IGZO semiconductor layer prove that a large amount of electrical current flows through the SiO_2_ insulator layer in case of that with a large IGZO semiconductor layer. Also, these results confirm the strong charge carrier transport in the MOSCAP with 36 mm^2^ IGZO layer at the accumulation states.

**References**

1. *Transport in Metal-Oxide-Semiconductor Structures,* Bentarzi, H., Springer, Berlin, **2011**.

2. Chiu, F. C. A Review on Conduction Mechanisms in Dielectric Films. *Adv. Mater. Sci. Eng*. **2014** (2014).

3. Choi, B. J., Chen, A. B. K., Yang, X., Chen, I. –W. Purely Electronic Switching with High Uniformity, Resistance Tunability, and Good Retention in Pt-dispersed SiO_2_ Thin Films for ReRAM. *Adv. Mater*. **23**, 3847 (2011).

4. Peng, H. Y., Li, G. P., Ye, J. Y., Wei, Z. P., Zhang, Z., Wang, D. D., Xing, G. Z., Wu, T. Electrode Dependence of Resistive Switching in Mn-doped ZnO: Filamentary versus Interfacial Mechanisms. *Appl. Phys. Lett.*, **96**, 192113 (2010).

5. Kim, C. Y., Lee, H. S., Woo, J. –K., Choi, C. K. Frequency-dependent Capacitance-Voltage and Conductance-Voltage Characteristics of Low-dielectric-constant SiOC(-H) Thin Films Deposited by Using Plasma-enhanced Chemical Vapor Deposition. *J. Kor. Phys. Soc*. **57**, 1976 (2010).
